# Corn starch-based coating enriched with natamycin as an active compound to control mold contamination on semi-hard cheese during ripening

**DOI:** 10.1016/j.heliyon.2019.e01957

**Published:** 2019-06-19

**Authors:** Lucía del Sol González-Forte, Javier I. Amalvy, Nora Bertola

**Affiliations:** a Instituto de Investigaciones Fisicoquímicas Teóricas y Aplicadas (INIFTA), (CCT La Plata CONICET- UNLP), Diag. 113 y 64, La Plata, Argentina; b Comisión de Investigaciones Científicas de la Provincia de Buenos Aires (CICPBA), Argentina; c Centro de Investigación y Desarrollo en Ciencia y Tecnología de Materiales (CITEMA), Facultad Regional La Plata (Universidad Tecnológica Nacional), 60 y 124, La Plata, Argentina; d Centro de Investigación y Desarrollo en Criotecnología de Alimentos (CIDCA), (CCT La Plata CONICET- UNLP-CICPBA), 47 y 116, La Plata, Argentina

**Keywords:** Food science, Food safety, Food microbiology, Microbiology, Natamycin, PVA, Polyurethane, Starch-based film and coating, Ripening, Microbiological contamination

## Abstract

The effectiveness of natamycin supported in corn starch-based films to control environmental molds (mainly *Penicillium spp*) activity that could colonize the surface of semi-hard cheese during ripening, was evaluated. The starch amount was maximized, and this was achieved by adding polyvinyl alcohol (PVA) and also polyurethane (PU) to the formulation. The PU acted as plasticizer and also provided functional groups that interacted with the natamycin and affected its diffusion. When 5 % PU was added, the natamycin migration of the coating doped with 1% natamycin was reduced by half. The natamycin distribution on both sides of the film was also evaluated, concluding that, in line with the reduced migration, when polyurethane is included, the formulation presents high hydrophobicity and natamycin is left with a preferential distribution towards the air face (exterior). For microbiological tests, microorganisms were isolated from cheese factories. Natamycin solutions showed inhibitory effect against environmental molds including *Penicillium spp*. Accordingly, films loaded with 0.1 % natamycin showed a significant inhibitory effect against *Penicillium spp.* The polymer combination in this work was optimized to obtain an active coating with good physicochemical properties and enriched with natamycin that has proven to be available for acting against molds and preferentially on the surface exposed to potential mold attack during ripening.

## 1 Introduction

Food safety is a global priority and one of the central issues of the current food legislation [1]. Natamycin is a polyene antifungal antibiotic produced by *Streptomyces natalensis* and plays a major part in the food industry to prevent yeasts and molds contamination of cheese and other non-sterile foods such as meat products (dried sausage or salami, for example) [2]. It is approved as a food additive in more than 40 countries and the FDA consider it as a GRAS (generally recognized as safe) product [3]. Natamycin inhibits vacuolar fusion [4, 5], not permeating but binding to ergosterol in the cytoplasmic membrane. As a result, natamycin is highly effective against molds and yeasts but not against bacteria and other microorganisms. Regarding application methods, it can be incorporated directly in a liquid matrix [6], or applied on solid surfaces by spraying, brushing or dipping. It is known that direct use of additives has limited benefits: the interaction with other components or additives in the food matrix could lead to a reduction in the active concentration of the antimicrobial component [7].

The two leading causes of food deterioration are microbial growth and oxidation reactions that take place on the product surface [8, 9]. Uncontrolled opportunistic microorganisms generate unwanted taste or odor, discoloration and can sometimes produce toxic secondary metabolites. This could be risky to most consumers, and it is also associated with a negative impact on product acceptance [10].

Incorporation of antimicrobials in film formulations can regulate the diffusion rate into the product, providing a way of maintaining high concentrations of the active ingredient on the surface [11]. The fact of restraining the additive to the surface also diminishes the interaction with food components and/or other additives [12]. As a consequence, films or coatings with antimicrobial activity are an auspicious form of antimicrobial delivery regarding food preservation [7, 13, 14, 15, 16].

Much research in recent years has focused on starch because of its low cost and easy availability from renewable sources. Unfortunately, pure starch films have limited application on account of the low water resistance and high brittleness of this material. Starch/polyvinyl alcohol (PVA) polymer combination is one of the most studied blends due to PVA's advantages such as high film forming capacity, good adhesive properties and high thermal stability. Nevertheless, numerous experiments have established that great amounts of PVA are required for the purpose of obtaining the desired starch/PVA film properties. In this work, a polyurethane (PU) of low glass transition temperature was incorporated as a plasticizer, since the purpose was to obtain films containing starch as the major component. Starch/PVA/PU films were obtained and studied in previous works, demonstrating that incorporating PU to the starch/PVA blends at around 15 wt. % represents a good method for improving the properties of the films [17] and that natamycin incorporation at 0.1 % causes minimal variations in the global characteristics and properties of films [18].

This paper focuses on the evaluation of the effectiveness of natamycin in starch-based films principally against *Penicillium spp* and typical mold that could colonize the surface of semi-hard cheese during ripening. To achieve this, microorganisms were isolated from two cheese productive establishments and microbiological tests were carried out.

## 2 Materials and methods

### 2.1 Materials

Commercial corn starch (Maizena Duryea^®^, Unilever Argentina S.A.), containing 0.11 g water, 0.006 g lipids, 0.003 g ash, 0.003 g proteins/g starch, and an amylose/amylopectin ratio of 25/75 (dry basis) was used [19]. Laboratory grade polyvinyl alcohol (PVA) was used (Sigma Aldrich, USA), with 98 % hydrolysis and a range of molecular weight (M_w_) from 13,000 to 23,000. The polyurethane (PU) was prepared from dicyclohexylmethane-4,4′-diisocyanate (H12MDI, Desmodur W, Bayer) and polypropylene glycol, M_n_ 2,000 (PPG2000, Voranol 2120, Dow) following a prepolymer process [17]. The cheese used was a semi-hard type Tybo (Cayelac, Argentina), containing 27 % total fat and 42–45 % moisture. The potato glucose agar, Mueller Hinton agar and Sabouraud Glucose agar used were Britania brand. Delvo^®^Cid XT1 natamycin was donated kindly by Harmony Group, "Food Specialties" Division.

### 2.2 Methods

#### 2.2.1 Preparation of film dispersion

The current investigation involved preparing the dispersions corresponding to the films starch/PVA/PU 70:30:0, 70:25:5 and 70:15:15. These were obtained following the procedure described by **González Forte et al.** [17]. Briefly, starch and PVA suspensions were prepared (starch-gelatinized dispersion by heating 3 wt.% of starch in water at 90 °C for 1 h and PVA by dissolving in water at 90 °C for 24 h, both with magnetic stirring) and a polyurethane previously synthetized in the laboratory was added to the mixture. For the polyurethane synthesis, PPG2000 and dimethylol propionic acid (DMPA, Sigma Aldrich, USA) were charged into a dried 1000 mL flask with a mechanical stirrer, thermocouple, condenser, sampling tube, inlet system for gases and pump feed inlet. While stirring, the mixture was heated to 90 °C, homogenized and bubbled dried N_2_ for approximately 60 min, followed by increasing the temperature to 98 °C and adding a mixture of H12MDI and dibutyltindilaurate catalyst (DBTDL, Sigma Aldrich, USA). After 2 h, the prepolymer was cooled to 45 °C and 2- hydroxy ethylmethacrylate (HEMA, Sigma Aldrich, USA) dissolved in acetone was added slowly and allowed to react for approximately 90 min. Then, the temperature was raised to 60 °C and kept constant until the isocyanate (NCO) content reached the desired value, determined using the conventional dibutylamine back-titration method [20]. The mixture was cooled to 55 °C and triethylamine (TEA, ADELFA S.A.) (in acetone) was fed in slowly over 50 min to reach the theoretical NCO value (ca. 4.7 %). After neutralization, the temperature was lowered to room temperature. An aqueous dispersion of PU was obtained by adding the PU prepolymer to water containing the appropriate amount of ethylene diamine (EDA, Sigma Aldrich, USA) to perform the chain extension reaction. The dispersion was performed at about 300 rpm in an ordinary glass reactor at 30 °C for 45 min. The resulting product was a stable dispersion with solid content of about 30 wt. % [21].

For the active films or coatings, natamycin water solution was prepared at 40 °C for 2 h in darkness and subsequently added to each mixture to obtain a final concentration of 1 % or 0.1 wt. %.

#### 2.2.2 Study of the natamycin distribution in the film

The distribution of natamycin on both sides of films (air and substrate) containing 1 wt. % natamycin was determined with infrared spectroscopy using an ATR accessory (Nicolet 380 spectrometer FTIR, Thermo Scientific, USA; ATR ZnSe IRE). The FTIR spectra were obtained by recording 64 scans between 4000–650 cm^−1^ with a spectral resolution of 4 cm^−1^. To achieve this, a comparison of the absorption spectra by FT-IR with the ATR accessory on both sides was made, particularly studying the contribution of the stretching band of the carbonyls from the conjugated esters of the lactone ring of natamycin at 1715 cm^−1^ [22,23]. Spectral data were acquired with EZ OMNIC software (Thermo Electron Corporation, USA), and applying baseline correction, ATR correction and noise reduction.

#### 2.2.3 Coating application on a model food

A commercial Tybo type semi hard cheese was chosen as a model system to evaluate natamycin effectiveness and behavior since cheese is one of the most popular products in which natamycin is applied worldwide. The choice of this type of cheese was made taking into account that this is a cheese with a homogeneously distributed humidity degree throughout its mass [24], allowing performing the tests with pieces simulating hole cheeses prior to ripening.

##### 2.2.3.1 Coating application and thickness measurement

Small pieces of Tybo cheese (3 × 3 × 1.5 cm^3^) were covered with the coatings to measure the final thickness obtained. In order to establish a clear difference between the film and the cheese surface, a concentrated aqueous solution of violet crystal dye was prepared and added to a starch/PVA/PU dispersion. Successive layers were made by immersing the cheese pieces in the dispersions for 5 s and immediately lifting them up to drain the excess. For drying, each piece was placed on a silicon plate that was put into a convection oven at 30 °C straightaway; flips were made every 10–15 min. This process was repeated until all the pieces were covered with three layers. Subsequently, a cut was made to each piece, and photographs were taken with a magnifying glass (10X magnification). The thickness was determined with Image-Pro Plus software. Triplicates were made for each sample.

##### 2.2.3.2 Natamycin diffusion assay

In the particular case of cheeses, the Argentine Food Code (AFC) establishes that natamycin is a permitted preservative on the surface that should not be detectable at 2 mm depth from the rind and, consequently, has to be absent in the mass [25, 26].

To carry out this test, starch/PVA/PU dispersions were prepared in 70:30:0, 70:25:5, 70:20:10 and 70:15:15 proportions, with an extra step to add the natamycin solution at 1 wt. %. Shortly after, the mixtures were concentrated by evaporation at 70 °C in a bath with mechanical stirring.

For this experiment, Tybo cheese was cut into cubes of 3 × 3 × 3 cm^3^. The cubes were immersed in the corresponding mixtures for 5 s and immediately lifted up to drain the excess, completing the process until covering the cubes with 3 layers. Triplicates were made for each sample.

The coated cubes were individually placed in plastic cheese bags, vacuum sealed with a Vacuum Saver kit (Food Saver, Tila, Italy) and then placed in a chamber at 10 °C for 75 days to simulate a ripening process.

To evaluate if natamycin diffused and penetrated more than 2 mm into the cheese mass, the technique of **de Ruig & van Oostrom** [27], which partially corresponds to **ISO 9233** [28], was followed. Briefly, after the exterior first 2 mm of each coated piece were discarded, the cheese was homogenized and a methanol:water (MeOH: H_2_O) extract was obtained. UV spectra were recorded for each sample, and analyzed particularly in the natamycin absorption wavelengths. Calibration curves corresponding to the minimum absorbance at 311.5 nm, and also for the maxima at 318 and 329 nm were conducted, choosing 311.5 and 329 nm wavelengths because of their best fitting. A solution of MeOH:H_2_O in 2:1 ratio was used as blank. This procedure was also used by **Vierikova, Hrnciarikova, & Lehotay** [29].

It has been reported that the application method has a great influence on diffusion: the interaction between cheese surface and coating has a direct influence on the migration efficiency of an active component [30, 31]**.**

#### 2.2.4 Microbiological tests

##### 2.2.4.1 Isolation of most representative colonies from cheeses

Three different strains were isolated from the surface of molded chesses from the “Cátedra de Agroindustrias, Facultad de Ciencias Agrarias y Forestales de la Universidad Nacional de La Plata” and from the “Colegio Agrotécnico Don Bosco Uribelarrea” of Buenos Aires Province. The surface of the cheese was scraped with a sterile swab, and then strains were isolated and grown in Sabouraud slanted agar at 28 °C for ten days [32]. The most representative strains were replicated on glucose-potato agar and Czapek-Dox agar. Macro and micromorphological studies were performed to the biggest colonies and microcultures.

##### 2.2.4.2 Sensitivity study of the films against Penicillium spp. and environmental contamination

Films of different composition were used: starch/PVA/PU 70:30:0, 70:25:5, 70:20:10 and 70:15:15. Three different assays were carried out:

1) Films inoculated with a drop of *Penicillium sp.* 1 suspension containing 1×10^9^ colony forming units (CFU)/mL and placed in Petri plates with 2 % agar;
2) Films inoculated with a drop of *Penicillium sp.* 1 suspension containing 1×10^9^ colony forming units (CFU)/mL and placed on a glass support in Petri plates with a paper soaked in sterile distilled water;
3) Films exposed for 1 h to the environment, without inoculation, in Petri plates with a paper soaked in sterile water.

Subsequently, the films were incubated at 10 °C for 45 days. All tests were performed in duplicate.

##### 2.2.4.3 Agar diffusion method

###### 2.2.4.3.1 Inhibitory activity of natamycin against isolated molds

For the purpose of evaluating the inhibition capacity of natamycin, three solutions were exposed to the different strains previously isolated and identified: *Penicillium sp. 1, sp. 2* and *sp. 3*, *Alternaria sp.*, *Fusarium sp.*, *Aspergillus Niger sp.*, *Mucor sp.* and *Cladosporium sp*. All fungi isolates were cultured on petri plates prior to microbiological essays.

To perform the Kirby-Bauer method Petri plates with Mueller-Hinton agar were used, and an inoculum containing 1 × 10^9^ CFU/mL of the strain was placed and spread homogeneously on the surface. Then, with the aid of a punch, 3 wells were made in the agar, in which natamycin solutions at 0.1, 0.2 and 0.3 % were placed. Finally, plates were incubated at 25–28 °C for 96 h and after that the inhibition zones were measured. Determinations were made in duplicate.

###### 2.2.4.3.2 Antimicrobial capacity of natamycin of loaded films

The agar diffusion test was also used to determine the antimicrobial effect of films loaded with natamycin against *Penicillium spp* 1, 2 and 3 isolated from cheese. Briefly, 0.5 μL of inoculum containing 1 × 10^9^ CFU/mL of each *Penicillium spp*, was spread on the surface of Petri plates containing Mueller-Hinton agar. Film disks (10 mm diameter) without natamycin, namely starch/PVA/PU 70:30:0, starch/PVA/PU 70:25:5 and starch/PVA/PU 70:15:15 (controls), and the same three films with natamycin (0.1 wt. %) were placed on plates previously inoculated. The plates were incubated at 28 °C for 96 h. The inhibitory activity was quantified by measuring the total diameter (disk plus inhibition zone). Determinations were made in duplicate.

#### 2.2.5 Statistical analysis of data

Data were analyzed through ANOVA (α = 0.05) and Tukey was the post-hoc test applied using InfoStat software [33]. Results are reported based on their mean and standard deviation.

## 3 Results and discussion

### 3.1 Study of the distribution of natamycin on both sides of the film and coating thickness measurement

**Fig. 1:**
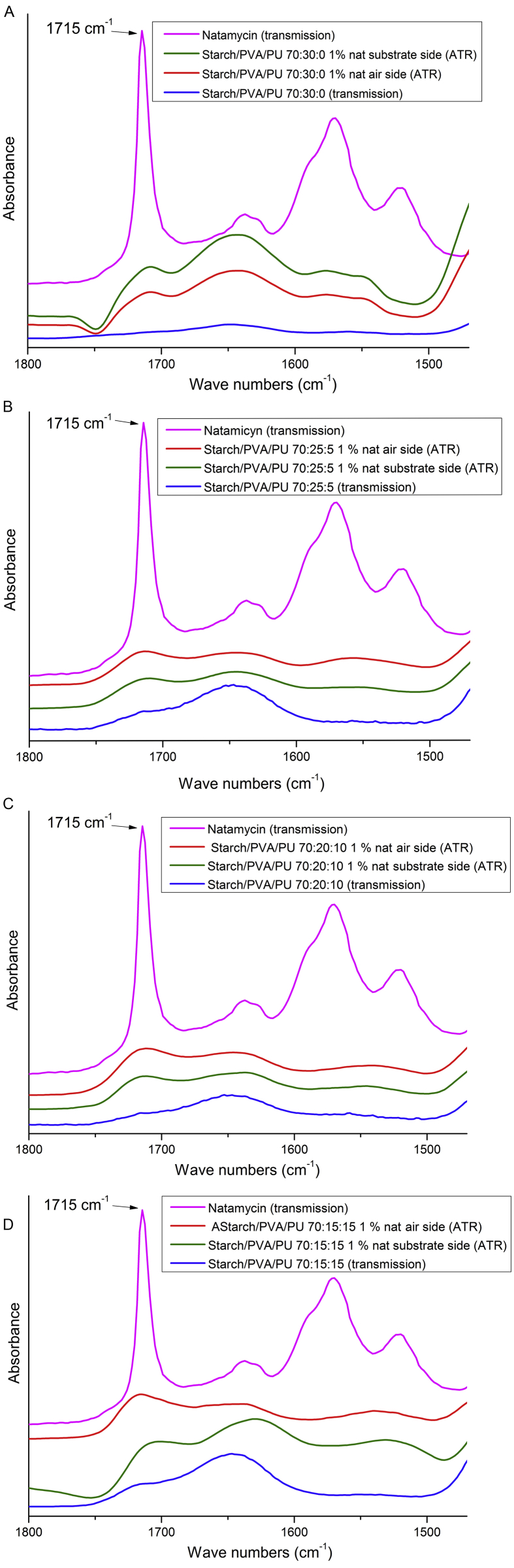
FTIR transmission spectra of pure natamycin and films containing 1 wt. % natamycin and ATR-FTIR spectra of substrate and air sides of the same film: (a) starch/PVA/PU 70:30:0; (b) starch/PVA/PU 70:25:5; (c) starch/PVA/PU 70:20:10; (d) starch/PVA/PU 70:15:15.

Fig. 1 shows the ATR-FT-IR spectra of starch/PVA/PU mixtures with natamycin. From the analysis for starch/PVA/PU 70:30:0 films (Fig. 1 A), it can be concluded that the amount of natamycin on the substrate side is greater than the amount in the air face. For films 70:25:5 and 70:20:10, the tendency is reversed (Figure 1 B and C). These films have a larger hydrophobic component due to the replacement of part of the PVA by PU, which leads to the natamycin being preferably distributed towards the air face. Although natamycin is amphiphilic, its structure has a more hydrophobic character, as indicated by the limited solubility in water [22, 34].

During the drying process for films starch/PVA/PU 70:30:0, the water evaporation front advances towards the air face. As a result, natamycin in inclined towards the substrate face. In contrast, when PU is present, the medium has a more hydrophobic characteristic and for that reason natamycin is better distributed, so when water is evaporated, natamycin could be left with a preferential distribution towards the air face. For the starch/PVA/PU 70:15:15 films (Fig. 1 D), there is a contribution to the absorption spectrum from the PU that masks natamycin. The modification of natamycin distribution in the film as a consequence of the incorporation of the PU could be useful to reduce the total content of the additive and dispose it selectively.

Coating thickness was measured and the results showed values within 55 ± 5 μm, which was aligned with the film thickness obtained by casting [17].

### 3.2 Diffusion assay of natamycin into the mass of semi hard cheese

**Fig. 2:**
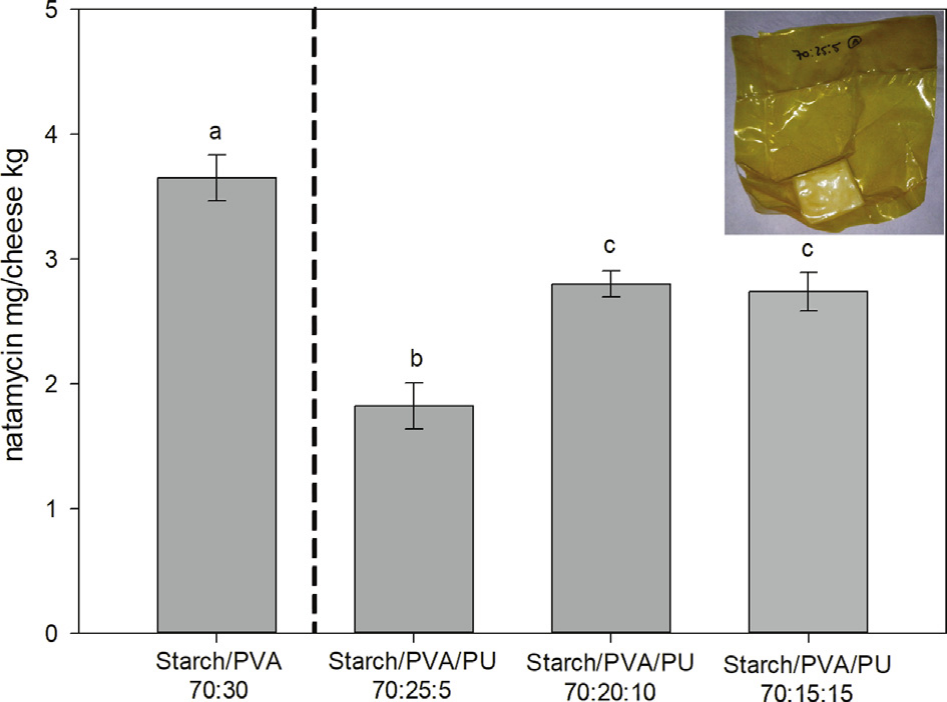
Content of natamycin in the cheese mass expressed as mg of natamycin/kg of cheese for cheese cubes coated with mixtures of starch/PVA/PU 70:30:0, 70:25:5, 70:20:10 and 70:15:15 with 1 wt. % natamycin. Equal letters indicate equal means (Tukey test, α = 0.05). In the upper right square, a photograph of a cheese cube coated with the formulation, vacuum packed.

Natamycin was found in the mass of every cheese and could be quantified (Fig. 2). Starch/PVA/PU coatings showed less natamycin release than the mixture without PU. Within the mixtures containing PU, the one with the lowest diffusion of natamycin was 70:25:5. Interactions between the film components and natamycin, and the amphiphilic nature of natamycin itself [22], determine in each case a different behavior in the diffusion of natamycin towards the cheese interior. The hydrophobic character of PU can limit the diffusion of natamycin which, despite being considered amphiphilic, has a more hydrophobic character due to its structure [34].

According to the AFC, natamycin should not be detected at 2 mm depth. Considering that the technique has a detection limit of 0.5 mg/kg, the results obtained with 1% wt. of natamycin (maximum detected value 3.65 mg/kg) and the fact that in this study the natamycin migration is over-estimated (ideal conditions for full migration: vacuum seal, time and temperature), it was decided not to proceed with the same study for 0.1 % natamycin, assuming that in this case both AFC conditions would be fulfilled.

### 3.3 Microbiological tests

#### 3.3.1 Strain isolation

An interesting practice in evaluating the effectiveness of a potential food coating is to carry out studies with microorganisms isolated directly from related environments: in this case cheese ripening rooms, processing rooms, or directly from the surface of molded cheeses [30, 35, 36, 37]. In this work the isolation of fungi from the surface of molded cheeses from ripening rooms resulted in the collection of three strains of *Penicillium*: *sp. 1*, *sp. 2* and *sp. 3*. **Basílico et al.** and **Hocking** [35, 38] showed that *Penicillium spp.* is a majoritarian mold that can be isolated from cheese surface. These microorganisms could be potential producers of mycotoxins dangerous to health [30, 35, 38].

#### 3.3.2 Sensitivity study of the films against Penicillium spp.

The results of the microorganism growth for films are presented in Table 1. The scale ranged between "-" and "+", where "-" symbolized no growth and "+" symbolized growth.

**Table 1:** Microorganism growth for films of starch/PVA/PU 70:30:0, 70:25:5, 70:20:10 and 70:15:15, exposed to the environment or inoculated with *Penicillium sp. 1* and *sp. 2*, incubated at 10 °C.

Films exposed to the environment for 45 days without inoculation				
	70:30:0	70:25:05	70:20:10	70:15:15
Films exposed to the environment, incubated at 10 °C	+	+	+	+
Films inoculated with *Penicillium spp 1 y spp 2* incubated for 45 days				
	70:30:0	70:25:05	70:20:10	70:15:15
On glass in plate, incubated at 10 °C	+	+	+	-
2 % agar, incubated at 10 °C	+	+	+	+

For the films exposed to the environment, only growth of environmental molds was registered, since there was no inoculation of the films. These environmental molds were isolated, analyzed and identified. The films incubated at 10 °C, presented *Penicillium spp.* development and additional contamination of environmental molds (Dematiaceae family, Deuteromycetes class), with the most frequent being the genera *Alternaria sp.* and *Cladosporium sp*. Less frequently, *Aspergillus Niger sp.*, *Fusarium sp.* and *Mucor sp*.

In general, all films containing starch showed a high fungal development after 45 days on agar, possibly because the agar constituted a wet surface in direct contact.

It should be considered that all samples were exposed to high humidity environments, either by the presence of a wet paper in the base of the boxes or by the agar itself, which combined with the temperature offered favorable conditions for the development of molds.

Ripening conditions are here proven favorable to mold development, and starch-based films are attractive substrates, which is why for this product it is crucial to incorporate an antifungal compound.

#### 3.3.3 Agar diffusion method

##### 3.3.3.1 Inhibitory activity of natamycin

**Fig. 3:**
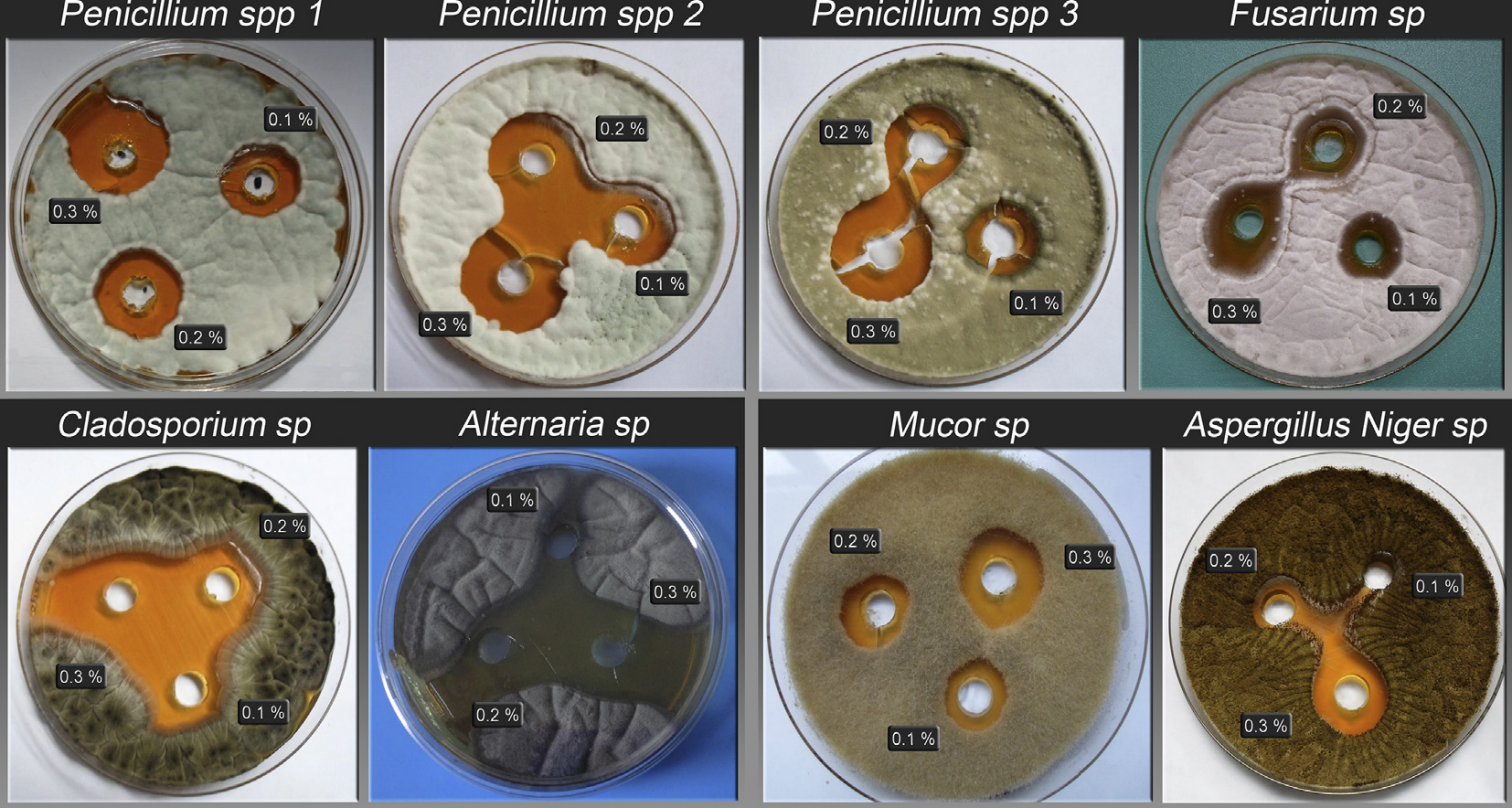
Inhibitory activity of 0.1, 0.2 and 0.3 % natamycin against *Penicillium spp 1*, *spp 2 and spp 3*, *Fusarium sp, Cladosporium sp, Alternaria sp, Mucor sp* and *Aspergillus Niger sp.*

The natamycin in concentrations of 0.1; 0.2 and 0.3 % had inhibitory activity against *Penicillium spp*. These concentrations are higher than the minimum proposed by **de Boer & Stolk-Horsthuis** [39] for inhibition effect with natamycin, and, it is important to outline that 0.1 % natamycin in the films is within the 1 mg/dm^2^ on cheese surface standard required by the AFC. The inhibition zones obtained against the different strains of *Penicillium spp.* of environmental molds studied after 96 h incubation are shown in Fig. 3.

It can be observed that the inhibition becomes greater for all samples as the amount of natamycin in solution increases; in all cases, the 0.3 % natamycin solution showed inhibition halos. For lower concentrations, the inhibition efficiency varied, probably because the differences between strains and some characteristics of the diffusing molecule as size, polarity and shape, and also interactions between the natamycin and the polymer chains, can affect the release of the agent.

##### 3.3.3.2 Evaluation of antifungal performance of natamycin loaded films

**Fig. 4:**
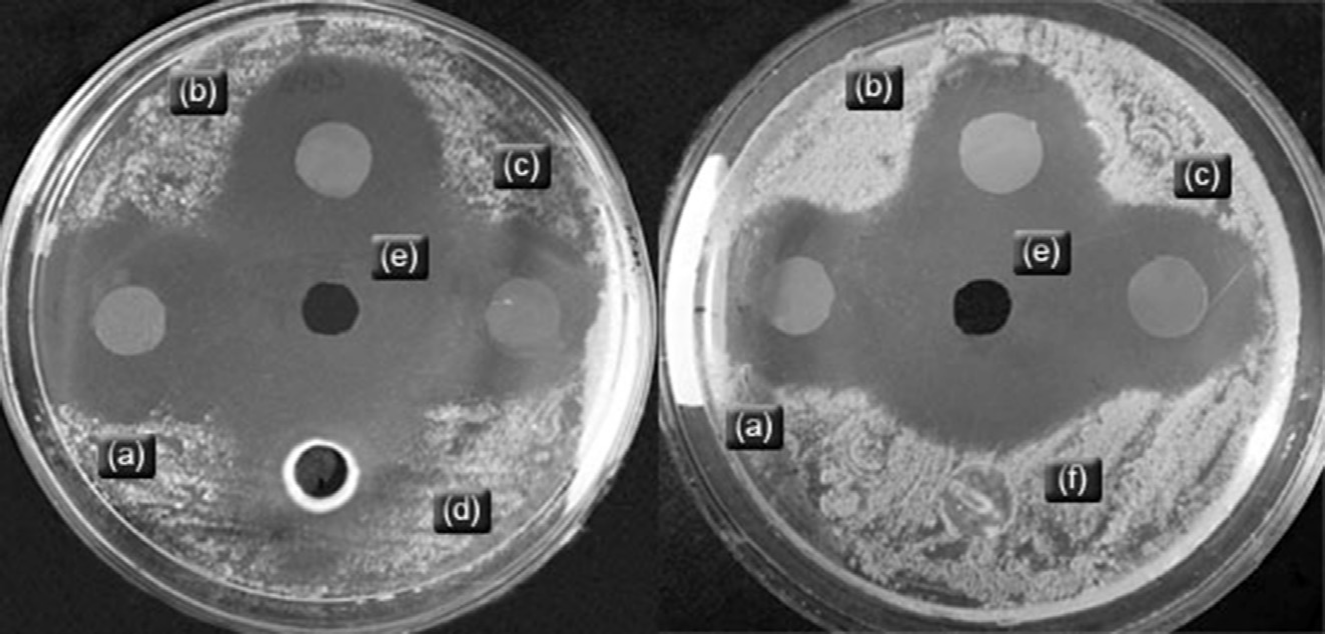
Inhibition halos of starch/PVA/PU films containing 0.1 wt. % natamycin: (a) 70:30:0, (b) starch/PVA/PU 70:15:15, and (c) starch/PVA/PU 70:25:5; (d) PU dispersion (50 % solid content); (e) natamycin aqueous solution 0.1 wt. % and (f) starch/PVA/PU 70:30:0 control film, against *Penicillium spp 1*.

The inhibition halos obtained for starch/PVA/PU films with or without 0.1% natamycin and natamycin solution and PU against *Penicillium spp.* are presented in Table 2. Fig. 4 shows an example of the inhibition zones against *Penicillium sp.* 1.

**Table 2:** Diameters of inhibition halos (mm) for starch/PVA/PU 70:30:0, 70:25:5 and 70:15:15 films with and without 0.1 % natamycin, PU dispersion and a solution of natamycin at 0.1 % wt.

Films and Controls	Inhibition halos in mm		
	*Penicillium sp. 1*	*Penicillium sp. 2*	*Penicillium sp. 3*
Starch/PVA/PU 70:30:0	0.0±0^a^	0.0±0^a^	0.0±0^a^
Starch/PVA/PU 70:25:5	0.0±0^a^	0.0±0^a^	0.0±0^a^
Starch/PVA/PU 70:15:15	0.0±0^a^	0.0±0^a^	0.0±0^a^
Starch/PVA/PU 70:30:0 0.1 % Nat	29.5 ± 1.5^b^	27.5 ± 0.5^b^	31±0^b^
Starch/PVA/PU 70:25:5 0.1 % Nat	26.5 ± 0.5^b^	30±2^b^	31±1^b^
Starch/PVA/PU 70:15:15 0.1 % Nat	29±1^b^	28.5 ± 0.5^b^	32.5 ± 1.5^b^
Polyurethane dispersion	40.5 ± 0.5^c^	49.5 ± 0.5^de^	50.5 ± 0.5^e^
Natamycin 0.1 % (solution)	44±3^cd^	44±2^cd^	43.5 ± 1.5^cd^

Values followed by the same letter are not significantly different according to the Tukey test.

These results showed that the 0.1 % natamycin solution and the pure PU applied in aqueous dispersion had a significant inhibitory effect on the 3 *Penicillium spp.* strains studied. The hydrophobic feature of the PU dispersion could limit the interaction with *Penicillium spp.* strains in aqueous suspensions, leading to an “inhibition halo”. Films loaded with 0.1 % natamycin showed a significant inhibitory effect, although the inhibition zones were smaller than those showed by natamycin solution; probably because natamycin is less available to act when incorporated into the films. This effect was the same statistically for all formulations of films versus all the strains.

A variety of works for soft, semi hard and hard cheeses with coatings or active films (synthetic or natural sources) containing natamycin can be found in the literature. In most cases, good results are obtained depending on the preservation times of the proposed cheese type [8, 9, 15, 30, 40]. **Hanušova et al.** [30] studied the release of natamycin from PVC films containing an 7.1 mg/dm^2^ of natamycin against isolated from the surface of a soft cheese (*Penicillium spp.* and *Cladosporium sp*., mainly), finding inhibition zones for all the strains against the active film. These authors also emphasize the importance of studying the difference between the effectiveness of natamycin in solution and natamycin contained in a polymer film.

There are several methods for applying natamycin in cheese preservation to prevent the growth of undesirable and/or potentially hazardous fungi on the surface. In the Argentinean industry, it is a common practice to spray the surface of the cheese with a natamycin solution, which means that there is no control over the amount of active compound per area unit, no control over the amount of natamycin that can migrate into the cheese and over the effectiveness of the treatment. **Reps, Jedrychowski, Tomasik, & Wisniewska** [41] studied the use of Delvo^®^Cid to protect semi hard cheeses against mold growth by different application methods: immersion of cheese in brine containing natamycin, immersion in aqueous suspension of natamycin (before or after brine), coating cheeses with polyvinyl acetate containing natamycin in layers with and without wax, packaging cheeses with films that were immersed in natamycin solution and packaging cheeses after immersion in natamycin solution with vacuum bags. The results showed that the content of natamycin is depleted over time, and that this loss depends on the application conditions of natamycin: the longer time of natamycin presence was obtained when one layer of wax was applied after 3 layers of polyvinyl acetate containing natamycin. **De Oliveira, De Fátima Ferreira Soares, Pereira, & De Freitas Fraga** [40] demonstrated, in agreement with these results, that the use of a layered coating containing natamycin results in a lower amount of natamycin in the cheese shell than if natamycin is directly applied in solution. Therefore, the efficiency of an antimicrobial film depends on: the amount of natamycin released, the contact between the cheese and the film, the barrier properties of the film and the temperature and humidity conditions in the ripening room.

## 4 Conclusions

Natamycin showed inhibitory effect against environmental molds including *Penicillium spp.* isolated from cheese factories. Films loaded with 0.1 % natamycin showed a significant inhibitory effect against *Penicillium spp*.

This study demonstrated that a corn-starch coating with starch percentage maximized to 70 % with PVA and PU as plasticizers and enriched with natamycin, could be applied as an effective coating to control environmental molds development on the surface of foods that require ripening like semi-hard cheeses. The polymers proportion was optimized to obtain an active coating with good physicochemical properties, and results showed that when PU is present in the formulation, natamycin adopts a preferential distribution towards the air side of the coating, being more available for acting against mold when applied on cheese surface.

## Declarations

### Author contribution statement

Lucia González-Forte: Conceived and designed the experiments; Performed the experiments; Analyzed and interpreted the data; Wrote the paper.

Javier Amalvy, Nora Bertola: Conceived and designed the experiments; Analyzed and interpreted the data; Contributed reagents, materials, analysis tools or data; Wrote the paper.

### Funding statement

This work was supported by CONICET Argentina, UNLP, and ANPCyT (Project PICT 2014–1785).

### Competing interest statement

The authors declare no conflict of interest.

### Additional information

No additional information is available for this paper.

## References

[1] Quintavalla S., Vicini L. (2002). Antimicrobial food packaging in meat industry. Meat Sci..

[2] El-Diasty E., El-Kaseh R.M., Salem R.M. (2008). The effect of natamycin on keeping quality and organoleptic characters of yoghurt. Arab J. Biotechnol..

[3] FDA (2003). 21CFR 172.55.

[4] Te Welscher Y.M., Ten Napel H.H., Balagué M.M., Souza C.M., Riezman H., De Kruijff B., Breukink E. (2008). Natamycin blocks fungal growth by binding specifically to ergosterol without permeabilizing the membrane. J. Biol. Chem..

[5] Te Welscher Y.M., Jones L., Van Leeuwen M.R., Dijksterhuis J., De Kruijff B., Eitzen G., Breukink E. (2010). Natamycin inhibits vacuole fusion at the priming phase via a specific interaction with ergosterol. Antimicrob. Agents Chemother..

[6] Gallo L., Pilosof A., Jagus R. (2006). Modelling Saccharomyces cerevisiae Inactivation by Natamycin in Liquid Cheese Whey.

[7] Ture H., Eroglu E., Ozen B., Soyer F. (2011). Effect of biopolymers containing natamycin against Aspergillus Niger and Penicillium roquefortii on fresh kashar cheese. Int. J. Food Sci. Technol..

[8] Var I., Erginkaya Z., Güven M., Kabak B. (2006). Effects of antifungal agent and packaging material on microflora of Kashar cheese during storage period. Food Control.

[9] Yangilar F., Oğuzhan Yildiz P. (2016). Casein/natamycin edible films efficiency for controlling mould growth and on microbiological, chemical and sensory properties during the ripening of Kashar cheese. J. Sci. Food Agric..

[10] Lund F., Nielsen A.B., Skouboe P. (2003). Distribution of Penicillium commune isolates in cheese dairies mapped using secondary metabolite profiles, morphotypes, RAPD and AFLP fingerprinting. Food Microbiol..

[11] Kristo E., Koutsoumanis K.P., Biliaderis C.G. (2008). Thermal, mechanical and water vapor barrier properties of sodium caseinate films containing antimicrobials and their inhibitory action on Listeria monocytogenes. Food Hydrocolloids.

[12] Campos C.A., Gerschenson L.N., Flores S.K. (2011). Development of edible films and coatings with antimicrobial activity. Food Bioprocess Technol..

[13] Costa M.J., Maciel L.C., Teixeira J.A., Vicente A.A., Cerqueira M.A. (2018). Use of edible films and coatings in cheese preservation: opportunities and challenges. Food Res. Int..

[14] Dos Santos Pires A.C., De Fátima Ferreira Soares N., De Andrade N.J., Da Silva L.H.M., Camilloto G.P., Bernardes P.C. (2008). Development and evaluation of active packaging for sliced mozzarella preservation. Packag. Technol. Sci..

[15] Fajardo P., Martins J.T., Fuciños C., Pastrana L., Teixeira J.A., Vicente A.A. (2010). Evaluation of a chitosan-based edible film as carrier of natamycin to improve the storability of Saloio cheese. J. Food Eng..

[16] Ollé Resa C.P., Gerschenson L.N., Jagus R.J. (2012). Effect of natamycin on physical properties of starch edible films and their effect on Saccharomyces cerevisiae activity. Food Bioprocess Technol..

[17] González-Forte L., Pardini O., Amalvy J. (2016). Starch/polyvinyl alcohol blends containing polyurethane as plasticizer. J. Compos. Biodegrad. Polym..

[18] González-Forte L., Amalvy J.I., Bertola N. (2019). Effect of natamycin on the physicochemical properties of corn starch based films and their effect on Penicillium spp. activity. Int. J. Polym. Anal. Charact..

[19] Ferrero C., Martino M.N., Zaritzky N.E. (1996). Effetct of hydrocolloids on starch thermal transition, as measured by DSC. J. Therm. Anal..

[20] Hepburn C., Science E. (1991). Analysis and characterization of polyurethane elastomers.

[21] Pardini O., Amalvy J. (2007). FTIR, 1H-NMR spectra, and thermal characterization of water-based polyurethane/acrylic hybrids. J. Appl. Polym. Sci..

[22] Brik H., Florey K. (1981). Natamycin. Anal. Profile Drug Subst..

[23] Atta H.M., Selim S.M., Zayed M.S. (2012). Natamycin antibiotic produced by Streptomyces sp.: fermentation, purification and biological activities. J. Am. Sci..

[24] Bertola N.C., Bevilacqua A.E., Zaritzky N.E. (1992). Proteolytic and rheological evaluation of maturation of Tybo argentino cheese. J. Dairy Sci..

[25] ANMAT, AFC, Capítulo XVIII- Aditivos Alimentarios (2014). Código Aliment.

[26] ANMAT, AFC, Capítulo VIII- Alimentos Lácteos (2014). Código Aliment.

[27] de Ruig W., van Oostrom J. (1987). Spectrometric and liquid chromatografic determination of natamycin in cheese and cheese rind. J. Assoc. Off. Anal. Chem..

[28] ISO, 9233-2 (2013). Cheese, Cheese Rind and Processed Cheese - Determination of Natamycin Content - Part 2: Highperformance Liquid Chromatographic Method for Cheese, Cheese Rind and Processed Cheese.

[29] Vierikova M., Hrnciarikova E., Lehotay J. (2013). Determination of natamycin content in cheese using ultra peormance liquid chromatography-mass spectrometry. J. Liq. Chromatogr. Relat. Technol..

[30] Hanušová K., Dobiáš J., Vold M. (2012). Assessment of functional properties and antimicrobial efficiency of polymer films with lacquer layer containing natamycin in cheese packaging. J. Food Nutr. Res..

[31] Bierhalz A.C.K., Da Silva M.A., De Sousa H.C., Braga M.E.M., Kieckbusch T.G. (2013). Influence of natamycin loading methods on the physical characteristics of alginate active films. J. Supercrit. Fluids.

[32] Pitt J.I. (1991). A Laboratory Guide to Common Penicillium Species.

[33] Di Rienzo J.A., Casanoves F., Balzarini M.G., Gonzalez L., Tablada M., Robledo C.W. (2018). Grupo InfoStat, FCA.

[34] Thomas A.H. (1976). Analysis and assay of polyene antifungal antibiotics: a review. Analyst.

[35] Basílico J.C., De Basílico M.Z., Chiericatti C., Vinderola C.G. (2001). Characterization and control of thread mould in cheese. Lett. Appl. Microbiol..

[36] Hanušová K., Štastná M., Votavová L., Klaudisová K., Dobiáš J., Voldrich M., Marek M. (2010). Polymer films releasing nisin and/or natamycin from polyvinyldichloride lacquer coating: nisin and natamycin migration. Effic. Cheese Packag..

[37] Moatsou G., Moschopoulou E., Beka A., Tsermoula P., Pratsis D. (2015). Effect of natamycin-containing coating on the evolution of biochemical and microbiological parameters during the ripening and storage of ovine hard-Gruyère-type cheese. Int. Dairy J..

[38] Hocking A.D. (1997). Understanding and controlling mould spoilage in cheese. Aust. J. Dairy Technol..

[39] de Boer E., Stolk-Horsthuis M. (1977). Sensitivity to natamycin (pimaricin) of fungi isolated in cheese Warehouses.pdf. J. Food Prot..

[40] De Oliveira T.M., De Fátima Ferreira Soares N., Pereira R.M., De Freitas Fraga K. (2007). Development and evaluation of antimicrobial natamycin-incorporated film in gorgonzola cheese conservation. Packag. Technol. Sci..

[41] Reps A., Jedrychowski L., Tomasik J., Wisniewska K. (2002). Natamycin in ripening cheeses. Pakistan J. Nutr..

